# An observational study of trait and state fatigue, and their relation to cognitive fatigability and saccade performance

**DOI:** 10.2217/cnc-2019-0003

**Published:** 2019-10-04

**Authors:** Marika C Möller, Jan Johansson, Giedre Matuseviciene, Tony Pansell, Catharina Nygren Deboussard

**Affiliations:** 1 Department of Rehabilitation Medicine, Danderyd University Hospital, 182 88 Stockholm, Sweden; 2 Department of Clinical Sciences, Division of Rehabilitation Medicine, Karolinska Institutet, 182 88 Stockholm, Sweden; 3 Department of Clinical Neuroscience, Eye & Vision, Karolinska Institutet, 112 82 Stockholm, Sweden

**Keywords:** cognition, fatigue, mild traumatic brain injury, neuropsychology, optometry, saccades

## Abstract

**Aim:**

Different fatigue measurements and their relation to saccadic functions were investigated in 15 patients with a mild traumatic brain injury (mTBI) and 15 orthopedic controls.

**Materials & methods:**

State fatigue was measured with the Fatigue Severity Scale and trait fatigue with the question on fatigue in the Rivermead Post Concussion Questionnaire and fatigability as decreased performance over time on a neuropsychological measure.

**Results:**

Patients with an mTBI scored significantly higher in state fatigue and showed more fatigability compared with the orthopedic controls. Among patients with mTBI, state fatigue correlated with prosaccade latency and cognitive fatigability, while trait fatigue correlated with anxiety and antisaccade latency and variability.

**Conclusion:**

This pilot study indicates that saccade measurements might, in the future, be useful in the understanding of fatigue and in the search for prognostic factors after mTBI.

Fatigue is a common symptom in many neurological conditions [1] and also one of the most frequently reported symptoms after a mild traumatic brain injury (mTBI) [2,3]. Although mTBI is associated with a good prognosis, fatigue is difficult to alleviate and it is often the main reason why patients fail to return to full-time work [4]. However, fatigue is not a specific complaint and multifactorial causes are plausible [1]. In addition, there is no gold standard for how to describe and measure fatigue. Several authors have stressed the importance of standardized taxonomy and assessment approaches that address distinct, objective aspects of fatigue and fatigability [1,5–7]. Mental fatigue, as measured by questionnaire, is a subjective experience [8]. Different self-reported scales get at different aspects of fatigue; the Fatigue Severity Scale (FSS), for example, asks for ratings of fatigue over a period of time, representing the person's predisposition for fatigue. This has been suggested to be described as trait fatigue. Fatigue at the moment, on the other hand, assessed with, for example, a visual analog scale, is described as state fatigue [9]. Another dimension of state fatigue after an mTBI could be valued by asking a person to compare the current level of fatigue with the experienced level of fatigue before the incident using, for example, the Rivermead Post Concussion Questionnaire (RPQ). This kind of fatigue is described as acquired fatigue in this paper. These are all self-rated methods to capture fatigue. At a functional level, fatigue can be operationalized to an inability to maintain performance over time. Cognitive fatigability is defined as an inability to maintain performance during a continued complex information processing task [5,6]. It requires, compared with a generally reduced sustain attention, a cognitively demanding task and a deterioration of performance over time [1]. It can be objectively quantified by computing decreased performance during time-on-task [10,11]. This method takes no account of the cause of fatigue but could serve as a method to objectify that the brain does not work effectively over time. In a previous study, our group has found that this could be a relevant method to capture fatigability in patients with mTBI [12,13] and other medical conditions [14,15].

Fatigue is sometimes a patient's sole complaint, but it can also be accompanied by a decline in cognitive performance [16,17]. In patients suffering from moderate-to-severe brain injuries, fatigue has been shown to be associated with reduced attention functions [18,19], executive functions [20] and reduced psychomotor speed [21]. There is often a lack of correspondence between self-perceived fatigue and cognitive measures after an mTBI [1,7,16,17,22]. One reason for the discrepancy might be that patients could, at least temporarily, compensate for cognitive impairment by increasing their efforts in the test situation [23,24]. As visual functions are fundamental to many neuropsychological tests measuring attention, visual functions might be part of both symptomatology and cognitive decline after a mTBI. Eye movements have been used to study brain functions in both healthy individuals and in patients with brain injuries [25] and the saccadic system has been suggested to be useful for investigating cognitive control [26]. A saccade is always preceded by an attentional shift from the foveally aligned object to the next object to be aligned. The perceptual shift is crucial for the saccadic system to choose where to look next while attention, on the contrary, does not depend on the saccadic system. However, the saccadic eye movements and attention work in a coordinated way [27]. All saccades have the same basic motor neural circuitry in the brain stem with additional cortical regions involved in the more neurally complex saccades [28]. For example, visually guided saccades, such as the prosaccades, are relatively simple and depend on external visual cues for the sensorimotor transformation. Volitional saccades, on the other hand, have been shown to activate prefrontal regions to a larger extent than visually guided saccades [29]. The antisaccade is an example of volitional saccades and depends on cognitive decisions to move the eye in the opposite direction of the cue, requiring inhibition and spatial memory. This is cognitively a more complex task and is considered to need frontal activation in the same way as the Stroop Color and Word Test [30]. Previous research has shown that mental fatigue is related to impairments on neuropsychological tests that require executive functions [5,12,19].

In this prospective explorative pilot study, we used three different measurements to examine the presence of fatigue: state fatigue or acquired fatigue, as self-rated change of fatigue compared with the level before the trauma; trait fatigue, as ratings of fatigue, which represents the person's predisposition for fatigue over period of time and cognitive fatigability, as decreased cognitive performance during time on task.

Our aim was to investigate different fatigue measurements and their relations to saccadic eye movements and attention functions in mTBI patients and patients with minor orthopedic trauma within 10 days of presenting at the emergency department (ED).

## Methods

### Participants

Eligible patients in the study were consecutive patients, between 18 and 40 years of age, presenting at the ED at Danderyd Hospital (Stockholm, Sweden) between January 2015 and April 2016 due to an mTBI to such an extent that CT was indicated.

The WHO Collaborating center of Neurotrauma Task Force on mTBI [31] definition, which is based on guidelines of Mild Traumatic Brain Injury Committee of American Congress of Rehabilitation Medicine [32], was used here: mTBI is an acute brain injury resulting from mechanical energy to the head from external physical forces. Operational criteria for clinical identification include:

- One or more of the following:
  - confusion or disorientation;
  - loss of consciousness for 30 min or less;
  - post-traumatic amnesia for less than 24 h, and/or other transient neurological abnormalities such as focal signs, seizure and intracranial lesion not requiring surgery.
- Glasgow Coma Scale [33] score of 13–15 after 30 min postinjury or later upon presentation for healthcare. The manifestations of mTBI must not be due to alcohol, drugs, medications, caused by other injuries or treatment for other injuries, caused by other problems (e.g., psychological trauma, language barrier or coexisting medical conditions) or caused by penetrating craniocerebral injury.

Patients were not eligible if the duration of loss of consciousness was uncertain, if they had contraindications to MRI, if they had a previously acquired brain injury, a progressive neurological disorder or another injury/illness with short expected survival, if they were dependent of help in daily living before the current damage, if they had severe visual impairment or were non-Swedish speaking.

The orthopedic controls (OC; n = 15) were in the same age span and consisted of patients of with minor traumatic injuries to the hand, arm, foot or leg with no need of surgical intervention. These patients were nonsystematically and intermittently included in the study during the same time frame as the mTBI patients.

A prior history, within 2 years of the study, of a traumatic head injury in a need of medical attention was an exclusion criterion for controls. See Table 1 for demographic information.

**Table 1.** Demographic information of the patients and controls.

	mTBI (n = 15)	Orthopedic controls (n = 15)	p-value
Age, mean (SD)	25.1 (6.5)	27.5 (7.4)	0.536
Gender, M/F (M%)	7/8 (47%)	11/4 (73%)	0.264
Length of education, mean (SD)	12.6 (1.8)	13.3 (1.8)	0.565
Premorbid IQ, mean (SD)	99.0 (7.8)	104.0 (9.8)	0.225
GCS 15, (n) GCS 14, (n)	14 1	N/A N/A	
HADS-D, median (range)	2 (0–5) (n = 14)	1 (0–11) (n = 14)	0.252
HADS-A, median (range)	5.5 (0–10) (n = 14)	4 (0–14) (n = 14)	0.186
Type of trauma: (n) (%)	Fall: 7 (47%) Bicycle: 2 (13%) HR injury: 2 (13%) Sports: 1 (7%) Other: 3 (20%)	Sports: 8 (53%) Bicycle: 1 (7%) MC injury: 1 (7%) Other: 5 (33%)	

Median, mean (range or SD) and p-values are presented.

Students t-test, Mann-Whitney U-test and Fisher's exact test with Freeman-Halton extension were used for comparison between the groups.

GCS: Glasgow Coma Scale; HADS-A: Hospital anxiety and depression scale – anxiety subscale; HADS-D: Hospital anxiety and depression scale – depression subscale; HR: Horseback riding; IQ: Intelligence quotient; MC: Motor cross; mTBI: Mild traumatic brain injury; N/A: Not applicable; OC: Orthopedic control; SD: Standard deviation.

This article is a separate report from a prospective controlled observational study on visual disturbances after mTBI. The power calculation was performed on an expected incidence of visual disturbances in 70% of mTBI patients and in 10% among the controls with a significance level of 5% (two-sided) and a power of 80%. With ten patients in each of the groups, these conditions were to be met. To compensate for possible dropouts, 15 patients in each group were enrolled.

### Study procedure

Giedre Matuseviciene (GM) or Catharina Nygren Deboussard (CND) checked the medical records at the ED at Danderyd Hospital daily. All injury-related data were collected from the medical records. Eligible patients with mild head injury or orthopedic trauma who met the inclusion criteria were approached at ED or, if discharged, contacted by phone within 1–3 days after the injury. Among the 129 patients who were asked for participation in the study (32 mTBI; 97 OC), a total of 99 people declined, 17 mTBI and 82 OC. The motives stated for not participating were a shortage of time and inconvenience of examination times.

Study participants received written information about the study by mail or at visits to the hospital. All study participants signed informed consent documents at first examination.

Assessments were done twice, at days 7–10 and at days 75–100. In this study only results from the first assessment are analyzed.

#### Assessment at days 7–10

Within 7 - 10 dyas after the trauma, demographic data at the initial interview, assessment of symptoms, cognitive and visual functions and structural MRI and resting-state functional MRI (rs-fMRI) were collected for the mTBI patients and OC. Data on rs-fMRI will be reported separately. The person who conducted the neuropsychological assessment collected demographic data. The visual medical history was taken at the time of the optometrist's investigation. Neuropsychological and visual assessments were performed at different times on the same day or consecutive days.

### Assessments

#### Visual functions

Licensed optometrists assessed visual functions using standard optometric clinical methods. These included assessment of monocular and binocular visual acuity near and far, refractive error, stereo acuity, near point of accommodation, facility (flexibility) of accommodation, near point of convergence with an accommodative target, nonstrabismic eye-turn (heterophoria), eye motility and fusional vergence. The results from these measurements have been reported earlier [34]. Identification of visual dysfunctions was based on established diagnostic criteria [35].

Saccadic stimuli were presented on a stimulus screen in front of the test subject at a 60-cm distance. Eye movements were recorded binocularly using an eye tracker (Tobii TX300, Tobii Corp, Stockholm, Sweden). Two test paradigms were applied to the test: visually induced prosaccades; latency and positional gain; antisaccades, latency and proportion of erroneous saccades. Latency pertains to the time from the onset of stimulus until the onset of the saccade. The positional gain refers to the accuracy of the saccade (saccade amplitude/stimulus amplitude). In both test paradigms, the stimulus was presented repeatedly (40-times) and mean value and standard deviation were calculated for each paradigm and patient.

The saccade stimuli consisted of a dot, 5 mm in diameter (subtending 0.5°). In the prosaccade paradigm, the participant fixated a centered cross and then refixated to a dot that appeared at 2, 4, 6 or 8° to the left or right of the cross. In the antisaccade paradigm, the participant viewed a centered cross and then rapidly looked in the opposite direction to that of a dot presented 8° to the left or right of the center.

#### Self-report questionnaires

The FSS was used to measure trait fatigue [36]. The questionnaire contains nine questions encompassing the behavioral consequences of fatigue and has been used to measure fatigue in TBI patients [37]. Each item is scored on a 7-point Likert scale. The final sum consists of the average for all the questions. A high score implies a higher level of fatigue and a cutoff score of 4 is usually used for stating a presence of fatigue [38,39].

The Hospital Anxiety and Depression Scale (HADS) was used to indicate the presence of depression and anxiety, of which fatigue is a core symptom [40]. A score of less than 8 points on the HADS depression subscale indicates no signs of depression, while a score of 8–10 indicates possible cases of depression and more than 10 points indicate a more definitive case of depression. The maximum summed scores of 21 for each subscale indicate severe depression/anxiety [41].

The RPQ was used for self-rated change in fatigue compared with levels prior to the mTBI [42]. RPQ is based on a Likert scale and includes 16 items with ratings from 0 to 4. A score of 1 means that the symptom has been present but is no longer a problem and a score between 2 and 4 indicates symptom severity from ‘mild to severe.’ Only the question regarding fatigue was included in the analyses and it was used to measure acquired fatigue in this paper.

#### Neuropsychological assessment

The Digit Symbol Substitution Test (DSST) [43] is a multifactorial subtest from the Wechsler scales, used for measuring psychomotor processing speed. The task is ongoing for 120 s during which the participant is expected to fill in symbols paired to digits. The score is the number of symbols correctly completed. Before being timed, the subjects are allowed one practice session of seven digits/symbols according to the manual [43]. Since psychomotor speed can be affected by learning capacity, incidental memory was measured. After four completed rows, the participants were requested to fill out the correct symbol under each digit twice [10]. In this study, Fatigability (DSST-f) was calculated by subtracting the score for the first half of the test (60 first seconds) from the last part of the test (60 last seconds). A negative score indicates a slower performance time during the latter part of the test. As the production of digits normally increases due to learning, a nonascending score could indicate fatigability [10]. DSST-f has previously been demonstrated to be a sensitive measure for fatigability, but not depression, in patients with mTBI [12].

The WAIS-III Digit Span from the WAIS-III was used for measuring verbal attention span with forward repetition of digits and verbal working memory with backward repetition of digits. The latter involves executive functions [30,43]. Sum scores for forward and backward repetition are presented separately.

The Ruff 2 & 7 Selective Attention Test (Ruff 2 & 7) was used to measures visual automatic detection speed (ADS) and accuracy, and controlled search speed (CSS) and accuracy. The test was administered as a continuous performance test, according to the guidelines in the manual [44]; the participants have to cancel numbers 2 and 7 in 20 different sections. In ten of the sections, two and seven are mixed with letters (ADS) and in the other ten sections, they are mixed with other numbers (CSS). The performance accuracy (emissions and commissions in relation to performance) is also counted automatic detection accuracy and controlled search accuracy (CSA), respectively. The subjects are allowed to practice before the timing begins – one section of ADS and one section of CSS. The total performance time is 5 min. High scores indicate better performance [44].

The Swedish Lexical Decision Test measures the estimated full-scale intelligence quotient (IQ) and was used to investigate the cognitive level between the study groups and was only used at the first assessment. Word knowledge and global cognitive functioning are generally associated. In this task, the subject must make a lexical decision by judging if target words are real or fictitious. The Swedish Lexical Decision Test has been demonstrated to explain 48% of the variance of full-scale IQ from the WAIS-R and 31% of demographic variables alone [45].

### Ethical approval

The study, which was carried out in accordance with the Helsinki Declaration guidelines, was approved by the regional ethics committee in Stockholm, Sweden. Verbal and written information about the study was given to all participants before giving informed consent.

### Statistical methods & data management

All data were anonymized in the database.

Comparisons of continuous, normally distributed data were performed by Student's t-test (age, length of education and DSST-f). The Mann-Whitney U-test was used for assessing categorical (RPQ-f, FSS, HADS, saccade functions, Ruff 2 & 7 accuracy measurements and incidental memory) and skewed variables.

Descriptive statistics were used to characterize the data. To evaluate hypotheses of variables in contingency tables, the Chi-square test was used. In the case of small expected frequencies, Fisher's Exact Test was implemented. To test independence between normally distributed variables (age, length of education and DSST-f), the Pearson correlation coefficient was used. Spearman's rank correlation was used for skewed variables and variables at the ordinal scale level (RPQ-f, FSS, HADS, saccade functions, Ruff 2 & 7 accuracy measurements and incidental memory). For effect size, Eta-square was calculated: Eta^2^ = Z^2^/N-1. Values between 0.00 and 0.04 equal a small group difference, 0.05–0.09 equal a moderate difference and ≥0.10 equal a large group difference. The study utilizes multiple hypotheses testing, where each hypothesis was analyzed separately and the existence of patterns in and the consistency of the results were considered in the analysis.

All analyses were performed by the use of statistical software, SPSS version 23 and a significance level of 5% was considered. In the case of a statistically significant result, the probability value (p-value) was given.

## Results

Fifteen patients with mTBI and 15 OC were analyzed. The first neuropsychological examination at the subacute stage was performed in the mTBI group with the median of 6 days after the injury (range 4–12 days) and 8 days (range 2–9 days) for the OC. Visual assessments and MRI were performed at different times on the same day or on two consecutive days. Two patients with mTBI had deviations in their computerized tomography of the brain; one had a small subarachnoid hemorrhage and the other one had a little subdural hemorrhage. Neither of them required surgery. There was no difference in age, gender, length of education or estimated IQ between the two trauma groups (Table 1).

### Comparison of self-reported fatigue & cognitive fatigability

The mTBI group scored significantly higher acquired fatigue (RPQ-f) (*U* = 58.5; p = 0.023) and showed more cognitive fatigability (DSST-f) (t[28] = -2.39; p = 0.024) compared with the OC. There was no significant difference between the groups regarding memory for the symbols (t[28] = 1.08; p = 0.291), in other words, incidental memory could not explain the difference in fatigability between the two groups. Trait fatigue, on the other hand, did not differ significantly between the mTBI group and the OC group (*U* = 64.5; p = 0.077). On average, the mTBI patients scored around the cutoff level for trait fatigue (FSS) and had mild problems with acquired fatigue (RPQ-f). The span, however, ranged from mild-to-severe symptoms. (Table 2).

**Table 2.** Self-rated fatigue, and fatigability among mild traumatic brain injury patients and orthopedic controls at the subacute stage and follow-up.

Questionnaires	mTBI (n = 15)	Orthopedic controls (n = 15)	Eta^2^	p-value
RPQ-f, median	2 (0–4)	1 (0–3)	**0.187**	**0.023**
FSS, median	4.0 (2.2–6.8)	2.8 (1.3–5.4)^†^	**0.112**	0.077
DSST-f, mean	-0.7 (3.2)	2.1 (3.2)	**0.176**	**0.024**

Median (range) or mean (SD) and effect size are presented.

Mann-Whitney U-test (questionnaires) and Student's – t test (DSST-f) for comparison.

† One missing.

DSST-f: Digit symbol substitution test-fatigability; FSS: Fatigue severity scale; mTBI: Mild traumatic brain injury; RPQ-f: Rivermead post-concussion questionnaire-fatigability; SD: Standard Deviation.

There were no correlations between age, length of education or predicted full-scale IQ and self-rated fatigue measurements (RPQ-f and FSS). Regarding cognitive fatigability (DSST-f), there were no correlations between these demographic variables for the mTBI group. In the OC group, however, length of education correlated positively with fatigability (DSST-f) (*r*_OC_.#x00A0;= 0.598; p = 0.019); the higher the education level the less fatigability.

### Depression & anxiety & their association to self-rated fatigue & fatigability

There was no difference between the groups regarding anxiety (*U_anx_* = 74.0, p = 0.186) or depression (*U_depr_* = 78.5, p = 0.252). Depression did not correlate with any of the fatigue measurements while there was a significant correlation between anxiety and FSS for the mTBI group (one missing) (*r*_mTBI_.#x00A0;= 0.716, p = 0.006) but not for the OC group (*r*_OC_.#x00A0;= 0.474, p = 0.074).

### Visual functions

Results of visual functions assessments from this study have been presented elsewhere [34]. The visual functions, convergence, accommodation and stereo acuity did not correlate with any of the fatigue measures.

### Saccade performance

There were no significant group differences regarding any of the saccade measurements, except for the variability of prosaccade latency (PSL) (*U* = 49.0; p = 0.024) and mean of the antisaccade latency (ASL) (*U* = 51.0; p = 0.031) (see Table 3). That is, the mTBI group showed more unstable prosaccade performance and they performed slower on the anti-saccade tasks.

### Self-rated fatigue & cognitive fatigability & their association to saccade performances

Different fatigue measures correlated to different saccade performances in the mTBI group. Acquired fatigue (RPQ-f) correlated positively with PSL (*r*_mTBI_.#x00A0;= 0.690; p = 0.006) for the patients with mTBI, but not with any of the other saccade measures. That is the higher the acquired fatigue, the higher PSL.

Trait fatigue (FSS) correlated with ASL (*r*_mTBI_.#x00A0;= 0.588; p = 0.035) and with the variability of ASL (*r*_mTBI_.#x00A0;= 0.637; p = 0.019) for the patients with mTBI. Meaning, the higher scores of trait fatigue the higher latency and higher variability in saccade performance. No correlations with prosaccades and FSS were found. No correlations were found between self-rated fatigue and saccades in any of the two control groups.

However, as there was a high correlation between FSS score and anxiety among the mTBI patients, partial correlation was performed. When controlling for anxiety the correlation between FSS and ASL did not remain significant for any of the groups; ASL: (*r*_mTBI_.#x00A0;= 0.300; p = 0.370; *r*_OC_.#x00A0;= -0.447; p = 0.126), and for variability of ASL: (*r*_mTBI_.#x00A0;= 0.067; p = 0.846; *r*_OC_.#x00A0;= 0.009; p = 0.978).

**Table 3.** Saccade functions subacute and at follow-up among mild traumatic brain injury patients and orthopedic and noninjured controls.

Saccade performance	mTBI (n = 15)	Orthopedic controls (n = 15)	Eta^2^	p-value
Prosaccade latency – mean	261 (222–371)	245 (222–270)	**0.101**	0.104
Prosaccade latency – SD	38 (25–125)	27 (18–45)	**0.181**	**0.024**
Prosaccade gain – mean	1.0 (0.9–1.1)	0.97 (0.8–1.1)	0.080	0.150
Prosaccade gain – SD	0.2 (0.1–1.4)	0.2 (0.1–0.3)	**0.113**	0.085
Antisaccade proper correct	0.8 (0.4–1.0)	0.8 (0.6–1.0)	0.005	0.701
Antisaccade latency – mean	347 (317–469)	326 (296–425)	**0.173**	**0.031**
Antisaccade latency – SD	43 (23–149)	37 (29–104)	0.057	0.227

Median (range) and effect size are presented.

Mann-Whitney U-test was used for comparison.

mTBI: Mild traumatic brain injury; SD: Standard deviation.

No correlations were found for cognitive fatigability.

### Self-rated fatigue & cognitive fatigability & their association to attention functions

There were no differences in attention functions between the groups (Table 4).

**Table 4.** Cognitive functions at first visit among mild traumatic brain injury patients and orthopedic and non-injured controls.

Neuropsychological tests	mTBI (n = 15)	Orthopedic controls (n = 15)	Eta^2^	p-value
Ruff 2 & 7 ADS (scores)	156.6 (28.8)	164.1 (28.0)	0.018	0.690
Ruff 2 & 7 ADA* (scores)	98.0 (90.0–100.0)	98.4 (92.3–100.0)	0.000	0.935
Ruff 2 & 7 CSS (scores)	126.8 (22.1)	133.5 (23.7)	0.023	0.630
Ruff 2 & 7 CSA* (scores)	94.3 (72.2–98.2)	90.2 (71.3–98.2)	0.025	0.412
DSST total (scores)	71.7 (10.0)	72.8 (15.9)	0.000	0.969
WAIS-III DSST memory* (score)	18 (8–18)	14 (7–18)	0.049	0.267
WAIS-III DS fw (scores)	8.3 (1.4)	8.9 (1.5)	0.057	0.321
WAIS-III DS bw (scores)	6.2 (2.1)	5.7 (1.8)	0.016	0.782

Mean (SD) is presented for nonskewed data and median (range) for skewed data, as well as effect size.

Student's t-test is performed and *Mann-Whitney U-test (two groups) for (skewed data) for comparison between the groups.

Ruff 2 & 7 ADA: Ruff 2 & 7 automatic detection accuracy; Ruff 2 & 7 ADS: Ruff 2 & 7 automatic detection speed; Ruff 2 & 7 CSA: Ruff 2 & 7 controlled search accuracy; Ruff 2 & 7 CSS: Ruff 2 & 7 controlled search speed; DSST total: Total score for WAIS-III Digit Symbol Substitution Test; WAIS-III DSST memory: Incidental memory for WAIS Digit Symbol Substitution Test; WAIS-III DS fw: WAIS-III Digit Span forward repetition; WAIS-III DS bw: WAIS-III Digit Span backward repetition.

SD: Standard Deviation.

#### Acquired fatigue (RPQ-f)

Acquired fatigue correlated negatively with cognitive fatigability (DSST-f) (*r*_mTBI_.#x00A0;= -0.527; p = 0.043), for the mTBI group; the higher self-rated fatigue the more fatigability. For the OC group, acquired fatigue correlated significantly with verbal attention span (*r*_OC_.#x00A0;= -0.515, p = 0.050).

#### Trait fatigue

Trait fatigue did not correlate with any of the attention measurements in the mTBI group but correlated negatively with psychomotor speed (DSST total score) (*r*_OC_.#x00A0;= -0.549; p = 0.034) and Ruff CSS score (*r*_OC_.#x00A0;= -0.625; p = 0.013) for the OC group.

#### Cognitive fatigability (DSST-f)

Cognitive fatigability (DSST-f) correlated positively with CSA (*r*_mTBI_.#x00A0;= 0.585; p = 0.022) for the mTBI group. The more fatigability the more errors on the Ruff 2 & 7 subtask demanding controlled attention functions. No correlations were found between fatigability and incidental memory, attention span or working memory in any of the groups.

## Discussion

In this study, we aimed to examine the presence and different aspects of fatigue among patients with mTBI and their association with saccadic eye movement performance and attentional function. As seen in other studies [2,46], patients with mTBI suffered from fatigue in the subacute stage, as they scored significantly more of acquired fatigue on RPQ compared with a control group having experienced orthopedic trauma. They also reached clinically significant trait fatigue according to the cutoff score on FSS. However, the FSS did not differ significantly between patients and OC.

**Figure 1.**
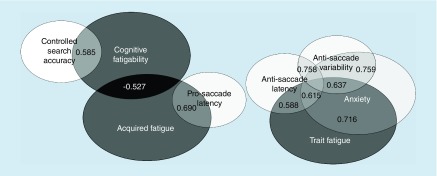
Significant correlations between the different fatigue measurements, saccade functions, attention and emotional state for the patients with mild traumatic brain injury.

Acquired and trait fatigue did not correlate within mTBI, indicating two separate mechanisms of fatigue. Moreover, they correlated differently to saccade performance. Acquired fatigue correlated to slower prosaccade performance while trait fatigue correlated to slower and unstable antisaccade performance. The correlation between prosaccade latency and acquired fatigue explained 48% of the variance. Anxiety, but not depression, was strongly related to trait fatigue and to antisaccade functions within mTBI (Figure 1) explaining 51 and 38% of the variance, respectively. Depression has commonly been associated with fatigue [40,47]. One possible reason for not finding any correlations between depression and fatigue at this early stage could be because patients scored relatively low on the depression scale in HADS while scoring higher on the anxiety scale, which made it easier to find a statistical relationship. It is reasonable to assume that patients after trauma are initially concerned with their symptoms and that depression might occur later in the process of nonrecovery. In a recent publication, Schiehser *et al.* [48] found a relation between anxiety and cognitive fatigue. Others have found altered saccade functions in anxiety [49,50]. Therefore, it is possible that antisaccade functions could mirror anxiety in an early stage in patients suffering from mTBI. We do, however, not know the causality, whether altered antisaccade functions are generating anxiety or if anxiety alters antisaccade functions.

Anxiety did not correlate with acquired fatigue or fatigability at the subacute stage (Figure 1). Instead, acquired fatigue correlated with fatigability, giving the assumption that a self-perceived change in the fatigue level at the early stage is more related to performance decrements. This is noteworthy as congruence between subjective and objective ratings is seldom reported. No associations were found between saccade performance, cognitive fatigability or attention. This should be expected, as attention does not depend on the saccadic system, rather fatigability seems more related to higher order cognitive functions.

As stated in earlier studies in this field, mental fatigue has been related to attention performance and reduced psychomotor speed [18,19,21] after an acquired brain injury or neurological disorders. The results of our study were in line with these findings. Even though the results were within the normal range and no group differences regarding attention functions and psychomotor speed emerged, cognitive fatigability among the patients with mTBI correlated significantly with more errors on the executive part of the Ruff 2 & 7 test. We have previously found that executive or complex tasks are prone to fatigability in mTBI patients [12] and other groups have found associations between fatigue, higher order attention [18] and executive functions [20]. The controlled attention aspects of the Ruff 2 & 7 test are associated with frontal networks in the brain [44] and fatigue has in several studies been related to frontostriatal networks [11,51,52].

Performance errors could also be viewed as unstable performance, which emerged on the saccade tasks. The saccade tasks were repeated several times and the patients with mTBI had significantly more variability in their performance level on the prosaccade tasks in comparison with the OC. An mTBI can lead to greater variability in performance [53] and performance variability might, therefore, be a more sensitive marker of a brain injury than the actual performance level *per se* in cases of minor injuries. In a previous study in our research group, variability on a vigilance task was associated with self-assessed state fatigue and linked to changes in neuronal networks [13].

Deficits in saccades have been suggested to be biomarkers of brain injury after mTBI and vision-related problems in clinical settings are discussed as potentially treatable deficits leading to headache and possible fatigue [54]. The different types of saccades might reflect different entities of brain functions. The same basic motor neural circuitry in the brainstem is used for all saccades with additional cortical regions involved in the control of different saccades. Antisaccades have been suggested to be an alternative marker for cognitive dysfunctions after an mTBI [54], but the results of this study could not support this. It is, however, interesting to note that the different fatigue measurements were linked to different saccade types. The prosaccades are expected to be more robust than the antisaccades as they involve a smaller network and are therefore not as sensitive. Antisaccades, on the other hand, are not reflexive to visual stimuli but depend on the internal decision to inhibit looking at the stimuli and instead move the gaze in the opposite direction. As inhibition needs the involvement of the frontal cortex, which is neurally more complex and under the influence of cognitive control, larger networks in the brain are used. Therefore, antisaccades might also be more sensitive to other ongoing cognitive processes and could be linked to nonbrain injury confounders as many other higher order cognitive processes. For example, sleep disturbances and anxiety can affect executive functions more than basic, simple cognitive functions [55,56]. The performance and variability of prosaccades might be a measure reflecting brain impact after an mTBI without disturbing confounders compared with antisaccades. Still, the differences in saccade functions between patients and controls were relatively small. These results differ from previous research by Heitger *et al.* among others [57], who found a strong association between saccade functions and remaining brain injury symptoms after concussion. Unlike Heitger's study that compared 36 symptomatic patients at a chronic stage with 36 nonsymptomatic, the patients in our study were consecutive patients in a subacute phase and they were also fewer, which could explain the different outcomes.

Our findings support the idea that there are several separate yet related mechanisms that contribute to fatigue [58] and we can generate multidimensional models of mechanisms of fatigue in mTBI by identifying these mechanisms. Our results indicate that acquired (state) fatigue after a brain injury is more related to fatigability and basal brain functions, while trait fatigue might be more related to the psychological reactions to the trauma (Figure 1). As there was no correlation between trait fatigue and acquired fatigue, the choice of assessment method is important to target relevant factors related to fatigue. The choice of assessment methods can, in the long run, affect the choice of interventions after an mTBI.

### Study limitations

The risks for type II error, as well as multisignificance, are high in a study with this small of sample size and the number of variables. The study should, therefore, be considered as hypothesis generating. Furthermore, study participants were strictly selected regarding age to avoid the effect of presbyopia on study results and cannot be generalized to a broader age group. Other limitations that could be discussed are the choices of questionnaires and assessments, for acquired fatigue (only one item) and cognitive fatigability (with a 2-min task). Although fatigue is a common complaint after mTBI and other neurological disorders, there is no consensus on the definition or how to assess fatigue. Therefore, the questionnaires and assessment used in this study should be considered as hypothesis generating in the clinically interesting but disparate area of fatigue.

## Conclusion

We found that fatigue was more pronounced in the mTBI group but that only acquired fatigue was related to cognitive fatigability and saccade performance while trait fatigue was more related to anxiety. This needs to be further investigated in fMRI studies and in larger prospective studies including neuropsychological measures. As fatigue is multidimensional in its nature [22,59,60] and different explanatory mechanisms are expected [61,62], different targets for interventions are thus likely.

## Future perspective

We still do not know the best model to explain, or method to assess, all of the underlying mechanisms behind fatigue. Imaging studies have found that frontostriatal networks are affected in conditions of fatigue [11,13,63–67]. Shallice *et al.* have found in lesion studies that superior medial frontal regions in the frontal lobe are important for what they call energization – keeping a stable performance level over time [68,69]. Future studies might show if energization and fatigability are also neuroanatomically related to state fatigue, prosaccade functions and fatigability in mTBI. One problem with cross-sectional studies is that we do not know if network dysfunctions have been dysfunctional for other reasons than the trauma. It is possible to follow the recovery process by designing an fMRI follow-up study on patients. If altered functions in cortical networks remain, even though patients have learned to compensate on cognitive tests, this would indicate a residual impact on the brain that cannot solely be explained by previously acquired dysfunctions.

**Summary points**

- Fatigue is one of the most frequent presenting symptoms after mild traumatic brain injury (mTBI) though other factors contributing to fatigue makes interpretation difficult.
- We aimed to examine the presence of different aspects of fatigue and their relation to saccadic and attention functions after mTBI.
- Fifteen patients with mTBI and 15 patients with minor orthopedic injury but no head trauma (orthopedic control, OC), all aged 18–40 years, were included.
- State fatigue after the injury was measured using the fatigue question in the Rivermead Post Concussion Symptoms Questionnaire. Trait fatigue was measured on the Fatigue Severity Scale, cognitive fatigability with WAIS-III Digit Symbol Substitution Test, attention functions with Ruff 2 & 7 and WAIS-III Digit Span, and saccadic performance by using an eye-tracking paradigm. Hospital Anxiety and Depression Scale controlled for depression and anxiety.
- Patients with an mTBI scored self-rated state fatigue significantly higher and showed more cognitive fatigability compared with patients in the OC group.
- Among patients with mTBI, state fatigue correlated positively with prosaccade latency and cognitive fatigability performance, while trait fatigue correlated with anxiety and antisaccade latency and antisaccade variability.
- Our findings support the theory of the multidimensional nature of fatigue but need to be validated in larger studies.
- Future research on fatigue should be conscious of which instrument is used to measure fatigue.
- Saccade measurements might be useful in the search for prognostic and modifiable factors for fatigue.
